# Establishment of a TaqMan qPCR method with MGB probe for the specific detection of BVDV field strains circulating in China

**DOI:** 10.3389/fvets.2025.1634429

**Published:** 2025-07-25

**Authors:** Lele An, Xiaoting Ren, Yetao Zhang, Shubin Zhang, Yongqing Zhao

**Affiliations:** ^1^Key Laboratory of Biotechnology and Bioengineering of State Ethnic Affairs Commission, Biomedical Research Center, Northwest Minzu University, Lanzhou, China; ^2^College of Life Science and Engineering, Northwest Minzu University, Lanzhou, China

**Keywords:** BVDV-1, BVDV-2, TaqMan MGB qPCR, clinical diagnosis, epidemiological investigation

## Abstract

Bovine viral diarrhea virus (BVDV), a highly mutable pathogen, poses a significant threat to the cattle industry in China. Therefore, the development of a rapid, sensitive, and specific diagnostic assay is essential for effective surveillance and control. In this study, a TaqMan real-time quantitative PCR (qPCR) assay utilizing a minor groove binder (MGB) probe was developed for the detection of BVDV, with a focus on strains currently circulating in China. Universal primers and an MGB probe targeting the conserved 5′ untranslated region (5′UTR) of both BVDV-1 and BVDV-2 were designed based on complete genome sequences available in GenBank. Following optimization of the reaction conditions, the assay demonstrated a detection limit of 1.265 copies/μL using a plasmid standard. The method exhibited high specificity for BVDV-1 and BVDV-2, with no cross reactivity observed with other common bovine pathogens. Intra- and inter-assay coefficients of variation were below 1.5%, indicating excellent repeatability and reproducibility. When applied to field serum samples collected from free-range cattle in various regions of China, the assay achieved a 100% concordance rate with a commercial reference kit (IDEXX RealPCR™ BVDV RNA Test). These results suggest that the established TaqMan MGB qPCR assay is a reliable and efficient tool for the detection and epidemiological investigation of BVDV-1 and BVDV-2 infections in cattle herds across China.

## 1 Introduction

Bovine viral diarrhea virus (BVDV), a member of the genus *Pestivirus* within the family *Flaviviridae*, is a significant pathogen affecting cattle worldwide. It poses substantial threats to the cattle industry due to its economic and health impacts (1–3). BVDV is a single-stranded, positive-sense RNA virus with a genome of approximately 12.3 kilobases (kb). The genome contains a single open reading frame (ORF), flanked by two untranslated regions (UTRs): the 5′UTR and 3′UTR. Phylogenetic classification of BVDV isolates primarily relies on sequence analysis of the 5′UTR and coding regions of viral proteins, including Npro (a viral protease), structural protein E2, and non-structural proteins NS4B and NS5A (4–9). Among these, the 5′UTR is the most widely used marker for genotyping due to its high conservation and discriminatory power. Based on the 5′UTR, different BVDV variants have been identified, leading to the current classification of BVDV into three genotypes: BVDV-1 (24 subgenotypes, 1a–1x), BVDV-2 (5 subgenotypes, 2a–2e), and BVDV-3 (HoBi-like pestivirus; 4 subgenotypes, 3a–3d) (10–15). Of these, BVDV-1 is the predominant genotype globally, accounting for approximately 88.2% of reported isolates (8).

The rapid expansion of the global cattle industry has increased awareness of the economic and clinical impacts of BVDV. Increasing evidence highlights that BVDV prevalence is associated with a range of economically important clinical diseases, including decreased reproductive performance and acute fatal hemorrhagic disease (16). Notably, BVDV can cross the placental barrier during early gestation, resulting in the birth of persistently infected (PI) calves, which serve as critical viral reservoirs (17). In China, BVDV-1 and BVDV-2 are considered the primary sources of infection in cattle herds, with their persistent circulation posing substantial risks to both animal health and the economic sustainability of the cattle industry (10, 18–22).

Current diagnostic approaches for BVDV include real-time quantitative PCR (qPCR), enzyme linked immunosorbent assay (ELISA), immunofluorescence assay (IFA), and viral isolation (6, 23, 24). Among these, qPCR demonstrates superior sensitivity for detecting acute infections. However, challenges arise from the high genetic variability of BVDV. Global genomic surveillance reveals extensive genetic divergence among BVDV variants, both between genotypes (e.g., BVDV-1 vs. BVDV-2) and within subgenotypes. In China, co-circulation of multiple BVDV-1 and BVDV-2 subgenotypes has been documented (4, 10, 22, 25–28). As more BVDV variants circulate within cattle herds, this increases the risks associated with sensitive detection, particularly in clinical cases involving serum samples.

To address these limitations, we developed a TaqMan MGB qPCR assay targeting the conserved 5′UTR region. This method was validated using serum samples collected from calves in diverse Chinese cattle herds between 2022 and 2024, with the aim of enhancing the detection of wild-type BVDV strains amidst evolving viral diversity.

## 2 Materials and methods

### 2.1 Serum sample collection

This study was approved by the Animal Ethics Committee of the College of Life Science and Engineering, Northwest Minzu University (Approval No. CLSEAEC 2020–003). A total of 174 whole blood samples were collected from free-range, unimmunized cattle herds in five Chinese provinces between 2022 and 2024. The distribution of samples across provinces was as follows: Henan (n = 28), Jilin (*n* = 36), Gansu (*n* = 54), Sichuan (*n* = 24), and Qinghai (*n* = 32).

Whole blood samples were transported on ice to the laboratory within 1 h of collection. The samples were allowed to clot at room temperature (25 ± 1°C) for 2–3 h, followed by centrifugation at 3,000 × g for 10 min at 4°C. The resulting serum was aliquoted into sterile cryovials and stored at −80°C until further analysis.

### 2.2 RNA extraction and cDNA synthesis

Total RNA was extracted from the serum samples using the MagMAX Viral/Pathogen Nucleic Acid Isolation Kit (ThermoFisher Scientific Inc.), following the manufacturer’s protocol. The RNA was then reverse transcribed into complementary DNA (cDNA) using the SuperScript IV First-Strand Synthesis System (ThermoFisher Scientific Inc.). The resulting cDNAs were stored at −20°C for subsequent use in the qPCR assays.

### 2.3 BVDV strain collection

Eight representative BVDV strains were isolated, identified, and preserved at the Laboratory of Biomedical Research Center, Northwest Minzu University. The strains used were as follows: 22Anhui-7 (GenBank: OQ338174.1), 22Gansu-F2 (GenBank: OQ338175.1), 22Sichuan-B8 (GenBank: OQ338177.1), 22NX-69 (GenBank: ON165517.1), 22AH-1 (GenBank: ON624286.1), 21SD-16 (GenBank: ON624287.1), BVDV1/GS (GenBank: PV806860.1), and 21NM-44 (GenBank: ON411191.1).

### 2.4 Other pathogens for specific testing

In consideration of the current epidemiological landscape of various viral pathogens in Chinese cattle herds, we focused on six common pathogens: foot-and-mouth disease virus (FMDV), bovine coronavirus (BCoV), bovine respiratory syncytial virus (BRSV), bovine parvovirus (BPV), classical swine fever virus (CSFV), and bovine parainfluenza virus 3 (BPIV3). Nucleic acids were extracted from clinical serum samples that tested positive for these specific pathogens. Viral DNA/RNA was isolated using the MagMAX Viral/Pathogen Nucleic Acid Isolation Kit, and the extracted nucleic acids were reverse transcribed into cDNA using the SuperScript IV kit. The resulting DNAs and cDNAs were stored at −20°C for subsequent analysis.

### 2.5 Designing a pair of universal primers and one MGB probe

**Figure 1:**
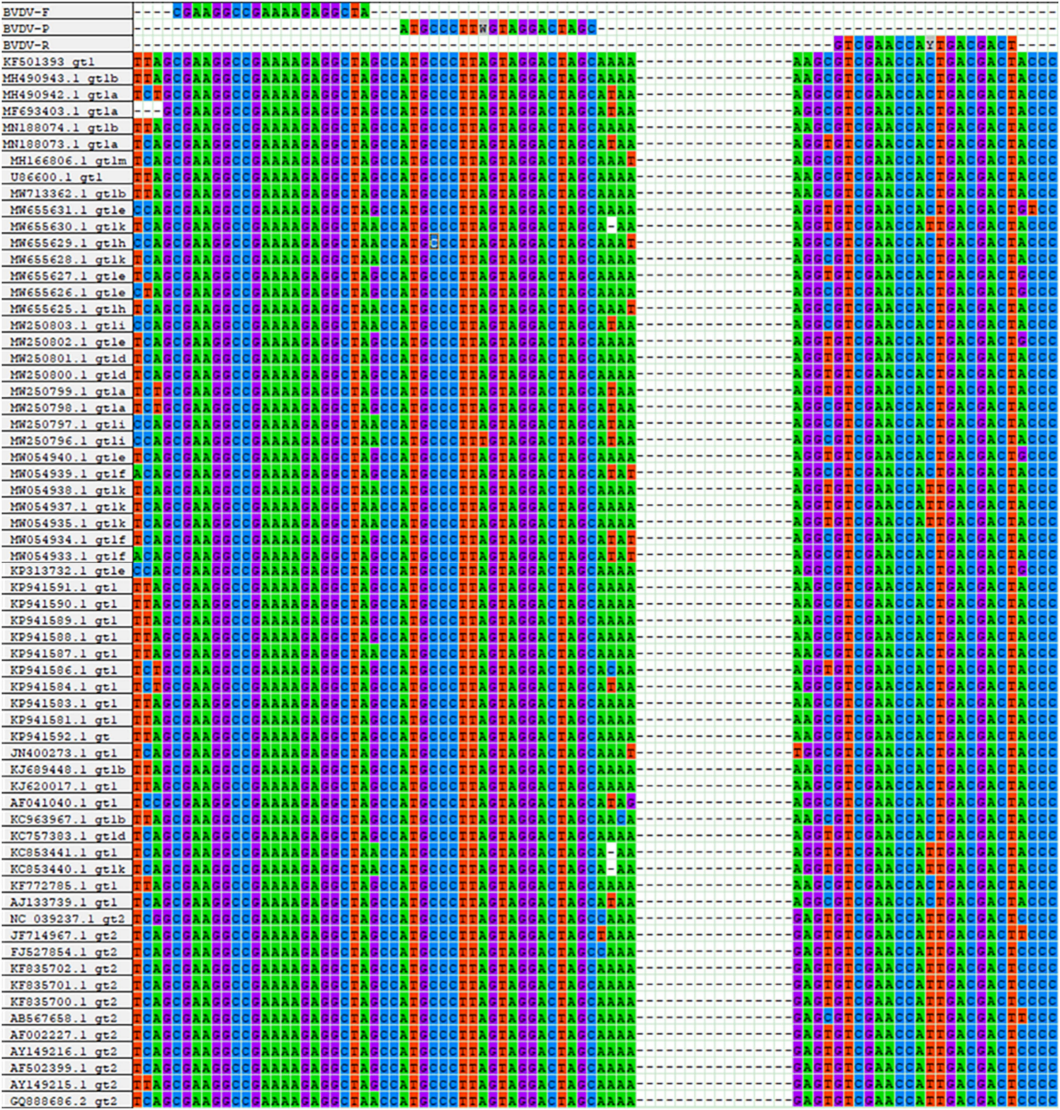
Sequence alignment information for primers and probes. The nucleotide bases color-coded as follows: cytosine (C) in blue, thymine (T) in red, adenine (A) in green, and guanine (G) in purple.

To identify relatively conserved regions in the BVDV genome, full genomic sequences of BVDV-1 and BVDV-2 strains, isolated from various countries and over different time periods, were retrieved from the GenBank database (Supplementary Table S1, DOI: 10.6084/m9.figshare.29143592). Multi-sequence alignment analysis was conducted using the ClustalW program within the MegAlign software. Based on the identified conserved regions in the BVDV genome, a pair of universal primers and an MGB probe were designed (Figure 1). All oligonucleotides were commercially synthesized by Tsingke Co., Ltd. (Xi’an, China).

### 2.6 Preparation of standard positive plasmids

Two representative BVDV strains, 21SD-16 (BVDV-1, GenBank: ON624287.1) and 22Sichuan-B8 (BVDV-2, GenBank: OQ338177.1), were maintained at −80°C in our laboratory. The target sequences derived from these two strains were amplified by PCR and cloned into the pMD18-T vector (TaKaRa, China). After verification by gene sequencing (Tsingke Co., Ltd., Xi’an, China), the two standard positive plasmids were designated as pMD18-T -BVDV1 and pMD18-T -BVDV2. The DNA copy number was calculated by the following formula: DNA copy number = (N × 6.02 × 10^23^ × 10^−9^)/(*l* × 660), where N is the amount of DNA in nanograms, and *l* is length of the plasmid in bp.

The concentrations of the pMD18-T-BVDV1 and pMD18-T-BVDV2 plasmids were determined to be 1.265 × 10^11^ copies/μL and 8.016 × 10^10^ copies/μL, respectively. The standard positive plasmids were serially diluted ten-fold, and the diluted plasmid standards with different copy numbers were used as templates to evaluate the performance of the BVDV TaqMan MGB qPCR assay.

### 2.7 Development of a TaqMan MGB probe-based qPCR assay for BVDV

To develop a robust detection assay, two plasmid standards were used as DNA templates in a TaqMan MGB probe-based qPCR assay. The reaction conditions were systematically optimized using response surface methodology (RSM) to establish the final qPCR protocol. Initial optimization involved fixing the MGB probe concentration at 0.2 μmol/L and evaluating the optimal primer concentration (0.35, 0.40, or 0.45 μmol/L). Based on the optimized primer concentration, we then assessed the optimal MGB probe concentration (0.25, 0.30, or 0.35 μmol/L). Finally, the annealing temperature (58°C, 60°C, or 62°C) was optimized using the selected primer and probe concentrations.

### 2.8 Construction of the standard curve

To establish the standard curve for the TaqMan qPCR assay with an MGB probe, a series of diluted vector solutions (ranging from 10^3^ to 10^8^ copies/μL) were used as DNA templates and amplified under the optimized detection conditions. To ensure reliability, each concentration was analyzed in triplicate.

### 2.9 Estimations for sensitivity and specificity of the assay

Based on the optimized detection system, we evaluated the sensitivity of TaqMan qPCR with MGB probes using a series of nucleic acid concentrations ranging from 10^0^ copies/μL to 10^4^ copies/μL as DNA templates. Additionally, sensitivity tests were conducted on plasmid pMD18-T-BVDV1, with concentrations ranging from 1.265 × 10^0^ to 1.265 × 10^6^ copies/μL, using a conventional PCR instrument. Deionized water was used as the negative control. To further assess the specificity of the detection method, we used TaqMan qPCR with MGB probes to detect BVDV and tested the cross-reactivity with other non-BVDV pathogens previously mentioned.

### 2.10 Inclusivity assessment of a TaqMan qPCR assay for BVDV detection

Eight representative epidemic BVDV strains—22NX-69, 22AH-1, 21SD-16, 21NM-44, 22Anhui-7, 22Gansu-F2, BVDV1/GS, and 22Sichuan-B8—were selected for inclusivity assessment. Viral RNA was extracted from each strain, reverse transcribed into cDNA, and subsequently analyzed using the established TaqMan MGB qPCR assay.

### 2.11 Assessment of repeatability and reproducibility of TaqMan qPCR for BVDV detection

To evaluate repeatability, the assay was tested using plasmid standards pMD18-T-BVDV1 and pMD18-T-BVDV2 at three concentrations (10^3^, 10^5^, and 10^7^ copies/μL). Both intra-assay (within-run) and inter-assay (between-run) precision were assessed by performing triplicate measurements. The arithmetic mean (x̄), standard deviation (SD), and coefficient of variation (CV) of Ct values were calculated to assess method stability and repeatability.

### 2.12 Detection of BVDV in clinical serum samples from multiple regions of China

To evaluate the clinical applicability of the TaqMan-MGB qPCR assay for BVDV detection, bovine serum samples collected from multiple provinces in China were analyzed. The performance of our assay was compared with a commercial BVDV qPCR kit (IDEXX RealPCR™, IDEXX Laboratories, Shanghai, China) using clinical samples. The positive coincidence rate was calculated as follows: (number of concordant positive results)/ [number of concordant positives + (TaqMan MGB qPCR negatives that were IDEXX method positives)] × 100%.

### 2.13 BVDV phylogeny based on TaqMan MGB qPCR detection

To determine the prevalent BVDV-1 and BVDV-2 genotypes across five Chinese provinces, one TaqMan MGB qPCR-positive amplicon from each province was randomly selected for direct sequencing (Tsingke Biotechnology Co., Ltd., Xi’an, China). Sequence alignment was performed using the ClustalW algorithm in MEGA 5.0 software, followed by phylogenetic tree construction using the neighbor-joining method with 1,000 bootstrap replicates.

## 3 Results

### 3.1 Development and validation of a TaqMan MGB probe-based qPCR assay

**Figure 2:**
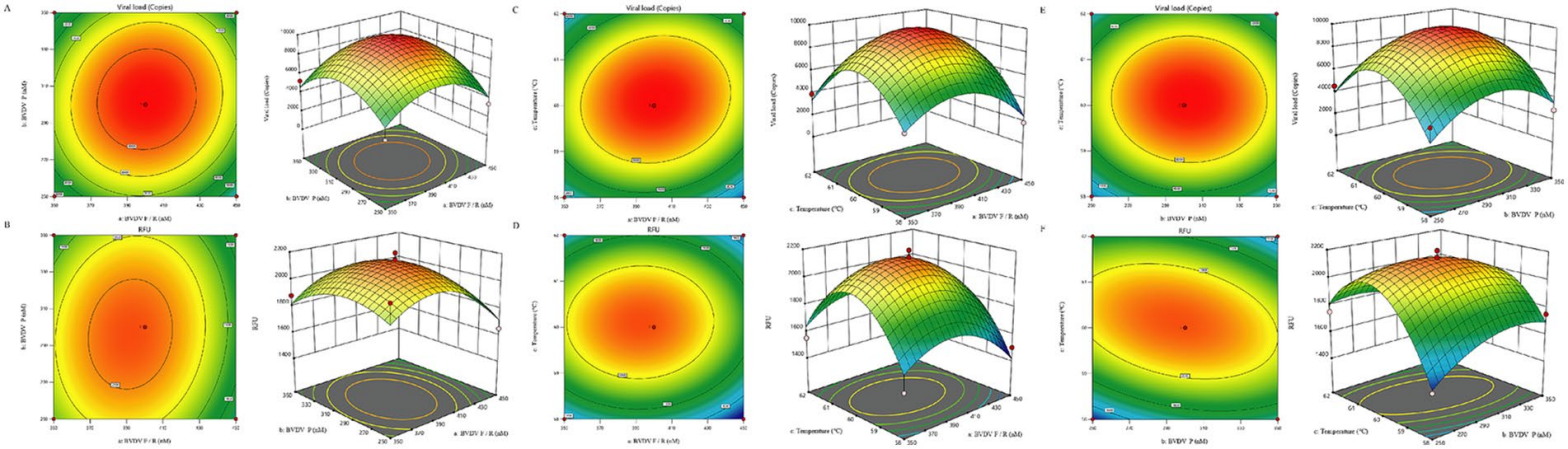
Response Surface Methodology (RSM) Optimization of BVDV TaqMan qPCR. **(A)** Effect of primer-probe concentration interaction on Ct values. **(B)** Effect of primer-probe concentration interaction on ΔRn. **(C)** Effect of primer-annealing temperature interaction on Ct values. **(D)** Effect of primer-annealing temperature interaction on ΔRn. **(E)** Effect of probe-annealing temperature interaction on Ct values. **(F)** Effect of probe-annealing temperature interaction on ΔRn.

Using Design-Expert 13 software (State-Ease Inc., USA), we optimized and analyzed the reaction conditions through response surface methodology (RSM). The model identified significant parameter interactions (*p* < 0.05), with the optimal conditions shown in Figure 2. The optimal amplification conditions were determined when the fluorescence signal intensity reached a plateau. The final parameters included a primer concentration of 0.42 μmol/L, a probe concentration of 0.30 μmol/L, and an annealing temperature of 60.2°C.

To validate the detection performance (sensitivity and specificity), we further optimized the TaqMan MGB qPCR assay under practical conditions. The final reaction conditions were as follows: 0.4 μmol/L primer, 0.3 μmol/L MGB probe, and an annealing temperature of 60°C. The 20 μL reaction mixture contained 10 μL of 2 × Pro Taq HS Probe Premix (Accurate Biotechnology, China), 1 μL template DNA, 0.8 μL each of upstream/downstream primers (BVDV-F/R, 10 μM), 0.6 μL MGB probe (BVDV-P, 10 μM), and ddH₂O to adjust the volume. The thermal cycling protocol included an initial denaturation at 95°C for 2 min, followed by 45 cycles of 95°C for 5 s and 60°C for 30 s.

### 3.2 Standard curve generation for BVDV quantification

**Figure 3:**
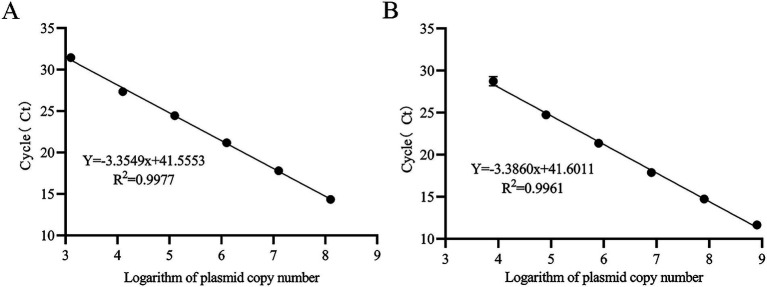
TaqMan MGB qPCR Standard Curve for BVDV Quantification. **(A)** Plasmid standard pMD18-T-BVDV1 (1.265 × 10^3^ ~ 1.265 × 10^8^ copies/μL) was used as the template. **(B)** Plasmid standard pMD18-T-BVDV2 (8.016 × 10^3^ ~ 8.016 × 10^8^ copies/μL) was used as the template.

Standard curves were generated using GraphPad Prism version 8.0.1 (GraphPad Software, USA). The logarithm of plasmid copy numbers (x-axis) was plotted against Ct values (y-axis), yielding linear regression equations:y = −3.355x + 41.555 (R^2^ = 0.998) and y = −3.386x + 41.601 (R^2^ = 0.996) for the two standards, respectively (Figure 3). The amplification efficiencies were 98.6 and 97.4%, which meet the MIQE guidelines criteria (R^2^ > 0.98, 90% < E < 110%). These results demonstrate a strong linear relationship between Ct values and logarithmic plasmid copy numbers.

### 3.3 Evaluation of TaqMan qPCR specificity and sensitivity for BVDV detection

**Figure 4:**
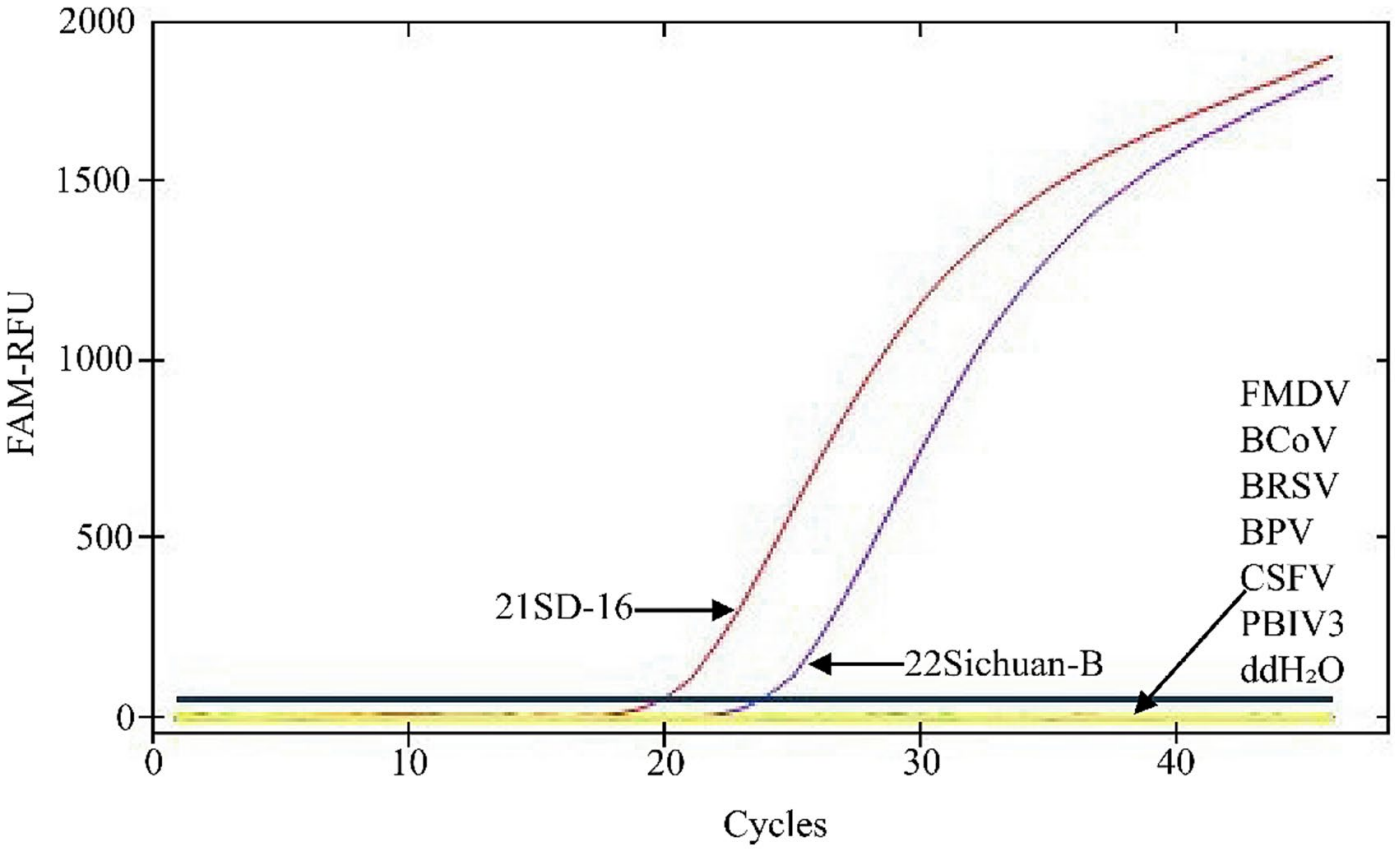
Specificity Test of the BVDV TaqMan MGB qPCR Detection Method. In the presented amplification graph, the x-axis represents the number of cycles, while the y-axis represents the relative fluorescence units (RFU). The amplification conditions for the following viral nucleic acids were tested: BVDV-1 (21SD-16), BVDV-2 (22Sichuan-B8), FMDV, BCoV, BRSV, BPV, CSFV, BPIV3, and a negative control (ddH₂O).

Detection specificity of the TaqMan MGB qPCR assay demonstrated that only BVDV-1(21SD-16) and BVDV-2(22Sichuan-B8) yielded characteristic sigmoidal amplification curves. No cross-reactivity was observed with other bovine pathogens, including FMDV, BCoV, BRSV, BPIV3, CSFV, BPV, or with the negative control (ddH₂O) (Figure 4).

**Figure 5:**
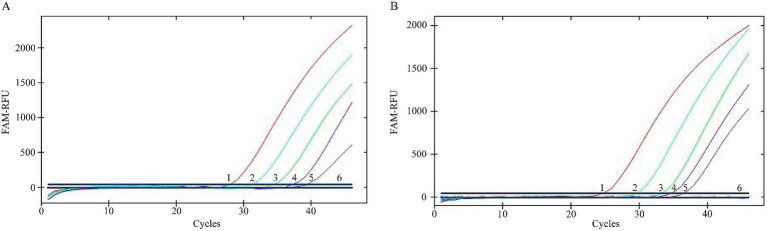
Analytical sensitivity evaluation of TaqMan MGB qPCR for BVDV Detection. The amplification curves for BVDV-1 and BVDV-2 were generated using plasmid standards pMD18-T-BVDV1 and pMD18-T-BVDV2, respectively. In panels **(A)** and **(B)**, the plasmid concentrations for curves 1 to 5 ranged from 1.265 × 10^4^ to 1.265 × 10^0^ copies/μL for BVDV-1 and from 8.016 × 10^4^ to 8.016 × 10^0^ copies/μL for BVDV-2. Curve 6 represents the negative control.

**Figure 6:**
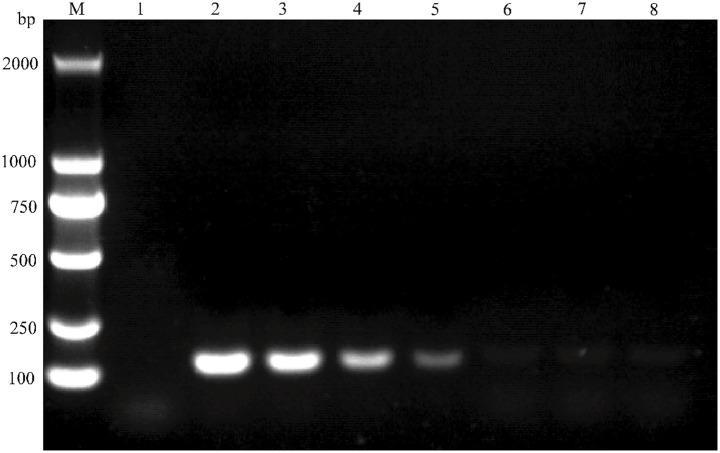
Sensitivity test for conventional PCR detection of BVDV. The target gene was amplified using plasmid standard pMD18-T-BVDV1 and analyzed by nucleic acid electrophoresis. In the electrophoresis pattern, Lane M: Molecular weight standard (DL 2000 DNA Marker); Lane 1: Negative control; Lane 2 ~ 8: Plasmid standard concentrations ranging from 1.265 × 10^6^ to 1.265 × 10^0^ copies/μL.

Assay sensitivity was evaluated using serial dilutions of the plasmid standards. The minimum detectable copy numbers were 1.265 copies/μL for pMD18-T-BVDV1 and 8.016 copies/μL for pMD18-T-BVDV2 (Figure 5). Both plasmid standards were tested in 20 replicates at their respective detection limits, yielding a 100% detection rate in both cases (detection rate >95%). Thus, the overall limit of detection (LOD) for the assay was established at 1.265 copies/μL. For comparison, conventional PCR using serially diluted pMD18-T-BVDV1 demonstrated a detection limit of 1.265 × 10^3^ copies/μL. This result confirms that the TaqMan MGB qPCR assay offers significantly enhanced sensitivity relative to conventional PCR (Figure 6).

### 3.4 Inclusion analysis

**Figure 7:**
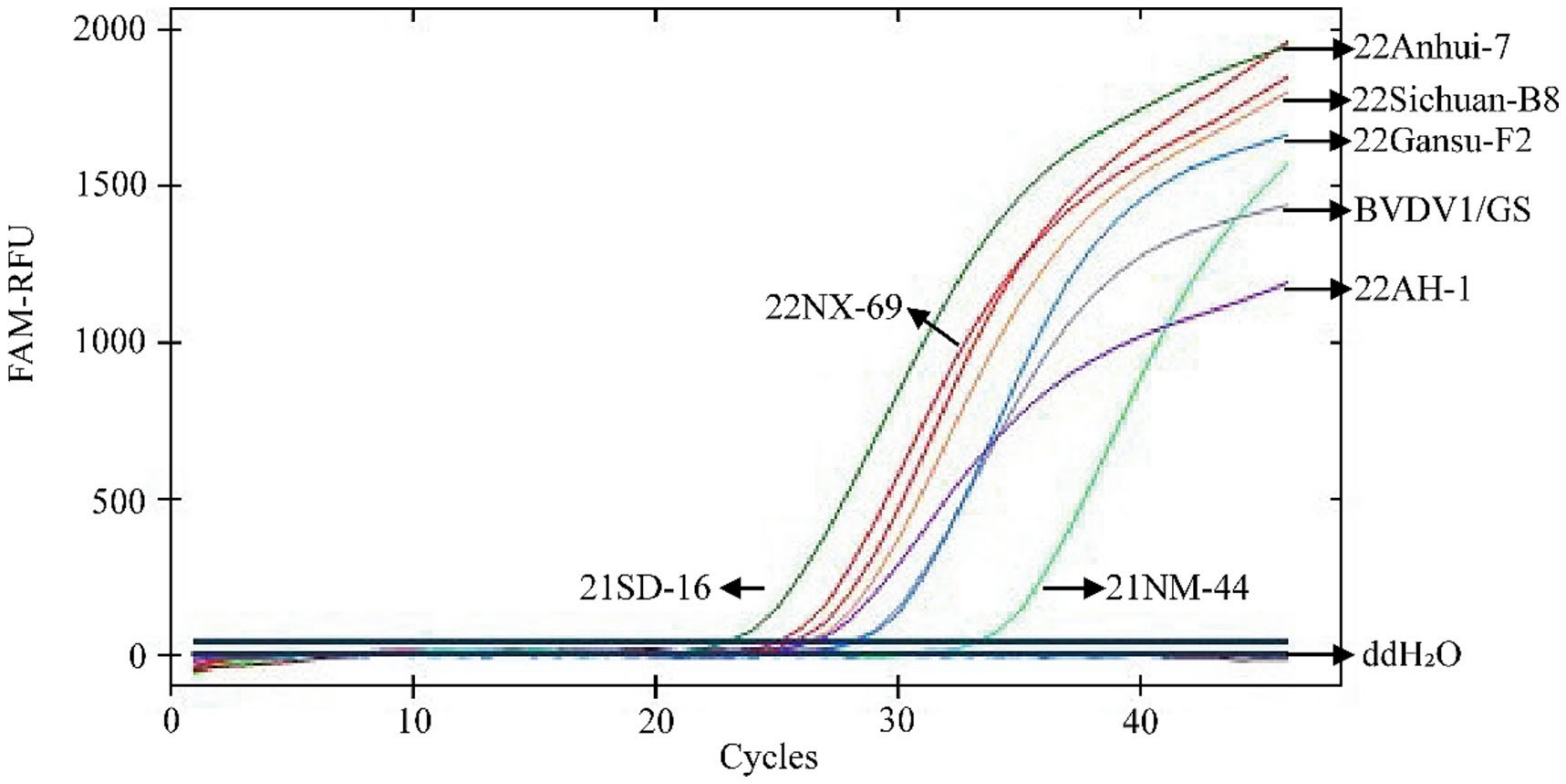
Inclusivity analysis of TaqMan MGB qPCR for BVDV detection. In the presented amplification graph, the x-axis represents the number of cycles, while the y-axis represents the relative fluorescence units (RFU). The amplification conditions for the following BVDV strains were tested: 21SD-16, 22NX-69, 21NM-44, 22AH-1, 22Anhui-7, 22Sichuan-B8, 22Gansu-F2, BVDV1/GS, and a negative control (ddH₂O).

The inclusive test (*n* = 3) demonstrated 100% detection of eight classical genotypic BVDV strains by the TaqMan^®^ MGB qPCR assay established in this study. The results are shown in Figure 7. This indicates that the newly developed assay is highly inclusive of the eight classical epidemic strains.

### 3.5 Repeatability analysis

Intra-assay and inter-assay repeatability tests were conducted using varying copy numbers of two plasmid standards as templates. The intra-assay coefficients of variation (CVs) ranged from 0.083 to 0.490%, while the inter-assay CVs ranged from 0.873 to 1.167%. All CVs were below 1.5% (Table 1), indicating high precision. These results demonstrate that the established qPCR assay exhibits excellent repeatability and stability.

**Table 1:** Intra-group and inter-group repeatability verification of TaqMan MGB qPCR methods.

Plasmid standards	Number of copies (Copies/μL)	Intra- group reproducibility tests			Inter-group reproducibility tests		
		Mean value x̄	Standard deviation SD	Coefficient of variation CV (%)	Mean value x̄	Standard deviation SD	Coefficient of variation CV (%)
pMD18-T-BVDV1	1.265 × 10^7^	17.80	0.040	0.226	17.99	0.175	0.971
	1.265 × 10^5^	24.15	0.118	0.490	24.01	0.257	1.071
	1.265 × 10^3^	31.45	0.026	0.083	31.49	0.275	0.873
pMD18-T-BVDV2	8.016 × 10^7^	14.80	0.062	0.417	14.91	0.161	1.077
	8.016 × 10^5^	21.62	0.090	0.415	21.77	0.192	0.880
	8.016 × 10^3^	28.40	0.065	0.231	28.81	0.336	1.167

### 3.6 Detection of clinical samples

**Figure 8:**
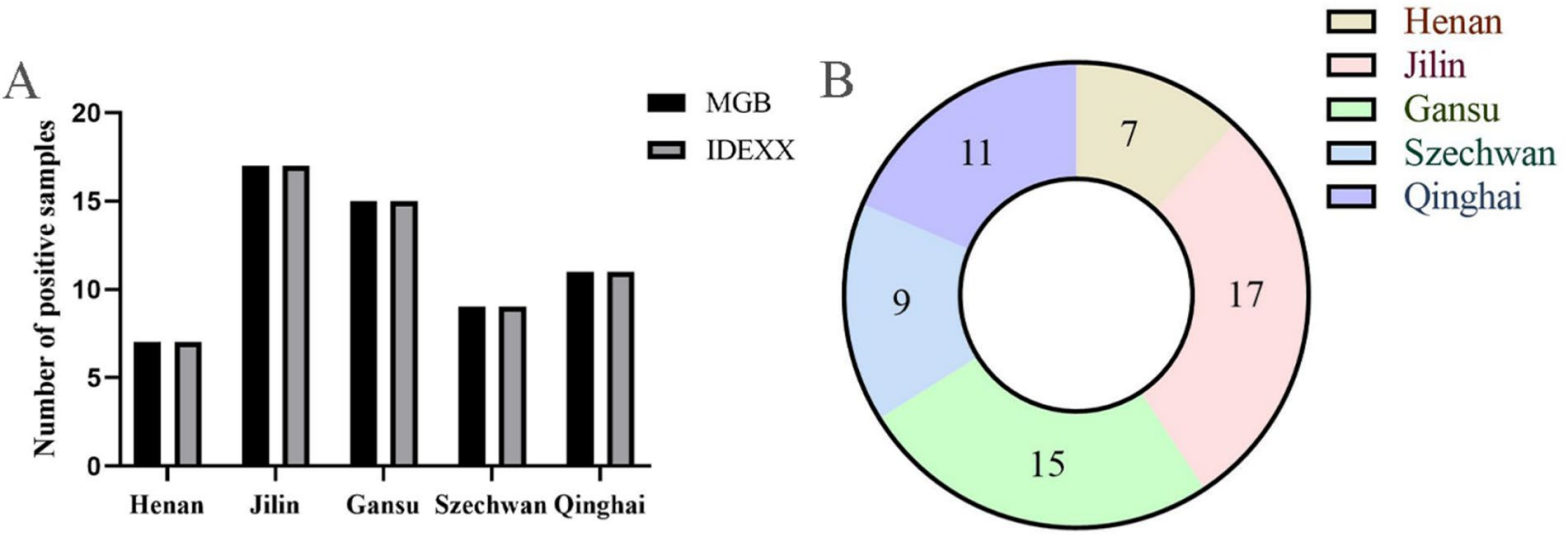
Detection of bovine serum clinical samples. **(A)** Comparison of TaqMan qPCR assay with commercial nucleic acid detection kit. **(B)** Distribution of BVDV positive clinical samples in different regions of China.

To further evaluate the clinical applicability of the assay for the differential diagnosis of BVDV-associated viral infections, a total of 174 bovine serum samples were tested using the established TaqMan MGB qPCR method. As shown in the bar chart (Figure 8), the results were fully consistent with those obtained using a commercial BVDV nucleic acid detection kit, yielding a 100% positive agreement rate. The overall positive detection rate among the tested serum samples was 33.9%. Region-specific positive rates were as follows: 25.0% (7/28) in Henan Province, 47.2% (17/36) in Jilin Province, 27.8% (15/54) in Gansu Province, 37.5% (9/24) in Sichuan Province, and 34.4% (11/32) in Qinghai Province. These results demonstrate that the developed qPCR assay possesses excellent clinical applicability for BVDV detection in diverse field settings.

### 3.7 Genetic evolution analysis of BVDV 5′-UTR gene

In the analysis of clinical samples, sequence homology and phylogenetic tree construction were performed on BVDV-positive samples collected from different regions. The results revealed that the Henan strain shared 97.44% nucleotide identity with the BVDV-1 HN1736 strain (GenBank accession no. MN442374.1). The Jilin strain exhibited 93.80% homology with the BVDV-1 NM2312 isolate (GenBank accession no. PP992316.2). The Qinghai strain showed 96.58% similarity to the BVDV-1 MRI3543 strain (GenBank accession no. LR822280.1). The Gansu strain demonstrated 98.32% homology with the BVDV-2 B9497 isolate (GenBank accession no. MH231134.1), while the Sichuan strain shared 97.48% identity with the same BVDV-2 isolate.

**Figure 9:**
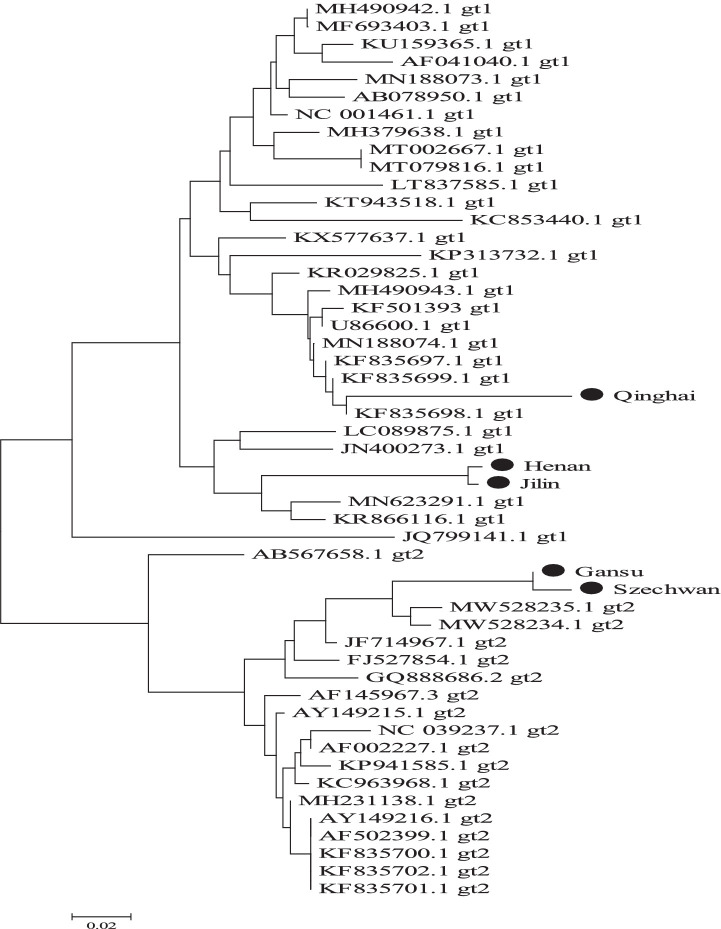
Genetic evolution of BVDV 5’-UTR gene in positive clinical samples from different regions of China.

Phylogenetic analysis further confirmed that the Henan, Jilin, and Qinghai strains clustered within the BVDV-1 genotype, while the Gansu and Sichuan strains grouped within the BVDV-2 genotype. Notably, the Henan and Jilin strains were closely related, forming a distinct phylogenetic branch, suggesting a potential shared lineage or transmission source. Similarly, the Gansu and Sichuan strains clustered together, indicating a close genetic relationship and suggesting possible interregional transmission between Aba Prefecture in Sichuan Province and the Gannan region in Gansu Province (see Figure 9).

## 4 Discussion

Since the first isolation and identification of BVDV in China in the 1980s, numerous outbreaks have been reported across various provinces (10). As a highly contagious disease that poses a significant threat to cattle health, BVDV has become endemic in many regions of China. The prevalent BVDV genotypes vary among countries and regions, and with the shift toward intensive, large-scale farming in China, the increased cross-regional movement of cattle has created favorable conditions for the spread of infectious diseases.

BVDV-infected animals may develop persistent infections (PI) and immunosuppression, both of which exacerbate disease severity and predispose affected herds to co-infections. Notably, PI cattle continuously shed the virus throughout their lives, serving as major reservoirs for BVDV transmission (29). Furthermore, PI animals are at risk of progressing to fatal mucosal disease (BVD-MD), which represents a severe threat to the sustainable development of China’s livestock industry (30, 31). Therefore, early detection and timely elimination of PI cattle are crucial for reducing the risk of BVDV outbreaks and ensuring effective BVDV control and eradication.

Due to the virus’s widespread distribution, high mutation rate, and genetic adaptability, numerous BVDV subtypes have emerged, enabling the virus to evade host immune responses (4). This genetic diversity necessitates continuous improvements in diagnostic techniques to facilitate broad-spectrum detection across genotypes and the early identification of animals in the latent stage of infection. Such advancements are vital for BVDV prevention and control, herd-level virus eradication, quality monitoring of veterinary biological products, and the reduction of economic losses in the cattle industry.

In this study, we developed a TaqMan MGB-based real-time PCR assay targeting the conserved regions of the 5′-UTR gene across BVDV-1 and BVDV-2 subtypes. Primers and MGB probes were designed based on sequence alignment of representative strains downloaded from NCBI. The assay was optimized for specificity and validated against common bovine pathogens, including FMDV, BCoV, BRSV, CSFV, BPIV3, and BPV. Only BVDV-1 and BVDV-2 strains yielded positive amplification signals, confirming the assay’s high specificity and lack of cross-reactivity. The limit of detection was determined to be 1.265 copies/μL, demonstrating superior sensitivity compared to previously reported methods. For example, a SYBR Green-based qPCR method developed by Hou et al. (32) had a detection limit of 5.2 copies/μL, and a TaqMan probe assay reported by Zhang et al. (33) showed a detection threshold of 10 copies/μL. The enhanced sensitivity of our assay is attributed to the use of MGB probes, which utilize fluorescence resonance energy transfer (FRET) principles and can detect single-nucleotide mismatches with high precision (34). Furthermore, MGB probes offer improved thermal stability, minimizing degradation under ambient conditions, a limitation common in traditional molecular beacon systems (35, 36).

Repeatability tests indicated a coefficient of variation below 1.5% for both intra- and inter-assay experiments, reflecting the method’s robust stability and reliability. Clinical validation with 174 field samples demonstrated a 100% concordance rate with results from a commercial BVDV nucleic acid detection kit, confirming the assay’s high accuracy and practical applicability. The ability to sensitively detect low viral loads and differentiate BVDV from other pathogens, combined with a rapid turnaround time (typically within 1–2 h), supports the assay’s suitability for clinical diagnostics and large-scale epidemiological surveillance.

Despite these advantages, certain limitations remain. Given the high genetic variability of BVDV, future improvements should focus on expanding primer and probe coverage to include a broader range of viral subtypes and emerging variants. Additionally, the sample size used for clinical validation was relatively limited. Subsequent studies involving larger sample sets from diverse geographic regions and herd types are needed to further assess the assay’s broad applicability and robustness.

## 5 Conclusion

In summary, this study developed a TaqMan MGB-based real-time PCR assay for the detection of BVDV genotypes 1 and 2. The assay demonstrated high specificity, excellent sensitivity, strong repeatability, and good clinical applicability. Combined with sequence analysis, it enabled precise differentiation between BVDV-1 and BVDV-2 without cross-reactivity with other bovine viruses. Clinical sample results showed strong agreement with those obtained from commercial kits, and phylogenetic analysis confirmed the assay’s universal detection capabilities for both major BVDV genotypes. This method provides valuable technical support for BVDV control efforts, including herd-level purification, clinical diagnostics, vaccine development, and epidemiological monitoring.

## Data Availability

The datasets presented in this study can be found in online repositories. The names of the repository/repositories and accession number(s) can be found in the article/supplementary material.
